# Supplementary material for: Atrial-like cardiomyocytes from human pluripotent stem cells are a robust preclinical model for assessing atrial-selective pharmacology
Source: EMBO Mol Med. 2015 Feb 19;7(4):394–410. doi: 10.15252/emmm.201404757 (PMC4403042; doi:10.15252/emmm.201404757)
Supplement: Supplementary file 4 — Supplementary Information [file emmm0007-0394-sd4.pdf]

Supplementary Information

Atrial-like cardiomyocytes from human pluripotent stem cells are a robust preclinical model for assessing atrial-selective pharmacology

Harsha D. Devalla[1]; Verena Schwach[1]; John W. Ford[2]; James T. Milnes[2]; Said El-Haou[2]; Claire Jackson[2]; Konstantinos Gkatzis[1], David A. Elliott[3]; Susana M. Chuva de Sousa Lopes[1,4]; Christine L. Mummery[1]; Arie O. Verkerk[5] and Robert Passier[1]

Table of Contents

Detailed Materials and Methods	-	3-7
Supplementary Tables S1-S11	-	8-16
Supplementary Figures S1-S8	-	17-24

Materials and Methods

Preparation of atRA:
atRA (Sigma) was diluted in DMSO to make a solution of 50 mmol/L which was further diluted in distilled water to have a stock solution of 0.0001 mol/L. atRA was used in a final concentration of 1 μmol/L in BPEL medium to direct atrial differentiation.

Human Material
Human fetal hearts of 12-15 weeks of gestation (n=3) were collected in phosphate buffered saline (PBS) and either fixed for immunohistochemistry or lysed for RNA isolation. Total RNA from adult heart was purchased (United States Biological, Massachusetts).

Collection and preparation of samples for microarray analysis:
Differentiated cells from Control and RA-treated conditions were collected at day 31 from two independent differentiations. Cells were dissociated with 10X TypLE^{TM} select (Life technologies) and filtered through falcon tubes containing 35um cell strainer lids (BD Biosciences). Cells were sorted for GFP on FACS ARIA^{TM} III (BD Biosciences). RNA was isolated (NucleoSpin RNA, Macherey-Nagel) from the recovered GFP^{+} and GFP^{-} fractions from both hESC-ventricular and hESC-atrial CMs.

For RNA isolation from human fetal tissue, atria and ventricles were separated from one heart at 15 weeks of gestation and lysed with TriZol (Invitrogen). Total RNA was extracted using NucleoSpin kit (Macherey Nagel).

Gene expression analysis:
Gene expression was determined on the HumanHT-12 v4 Expression BeadChip at ServiceXS, Leiden, The Netherlands. Biotin-labeled cRNA samples were amplified with Illumina® TotalPrep^{TM}-96 RNA Amplification Kit. Hybridization and scanning were performed according to standard Illumina protocols. Expression profiles were established from two independent samples each for GFP+ and GFP- populations of hESC-ventricular and hESC-atrial differentiations, isolated at day 31. Data normalization and analysis was performed using GeneSpring v12.6 (Agilent Technologies). Genes differentially expressed in both the replicates and satisfying the fold change cutoff of >2.0 were selected for further analysis. ConsensusPathDB-human (http://cpdb.molgen.mpg.de/) web server was used for gene ontology analysis. Microarray data has been deposited in the GEO database with the accession number GSE61154.

Quantitative PCR (qPCR)
For assessing gene expression by qPCR, cDNA was synthesized from approximately 1μg of RNA obtained from three independent differentiations (iScript cDNA Synthesis Kit, BIO-RAD). Target identification was performed using SYBR Green (Applied Biosystems, Life technologies) and detected by CFX96 Real-Time PCR system (Bio-Rad). Each reaction was performed in triplicates and non-template reaction (replacing cDNA with water) was used as negative control. The cycling parameters were 95°C for 3 minutes followed by 95°C for 10 seconds, 60°C for 10 seconds and 72°C for 30 seconds for 40 cycles. Absolute mRNA levels were normalized to human acidic ribosomal phosphoprotein (hARP) which was used a reference. Primer sequences are enclosed (Table S10)

Immunocytochemistry:
EBs plated on plastic coverslips were fixed in 2% paraformaldehyde (PFA) followed by permeabilization with 0.1% Triton-X 100 (Sigma Aldrich) in phosphate buffered saline (PBS). Blocking was performed with 4% normal swine serum (Dako) in PBS and following primary antibodies were used: NKX2.5 (clone H-114, Santa Cruz); ACTN2 (clone EA-53, Sigma); Troponin I (clone H-170, Santa Cruz); COUP-TFI (H8132, Perseus proteomics or D4H2, Cell Signaling); COUP-TFII (H7147, Perseus Proteomics); Primary antibodies were detected using secondary antibodies conjugated to fluorophores, Alexa Fluor 647 or Cy3 (Anti-Mouse Cy3, Anti-Rabbit Cy3- Jackson Immunoresearch; Anti-Mouse Alexa 647, Anti-Rabbit Alexa 647- Life technologies).

Nuclei were counterstained with DAPI (Life technologies). Images were captured using Leica TCS SP8 microscope (Leica Microsystems, Germany) and acquisition was performed with LAS AF software (Leica, Germany).

Immunohistochemistry of human heart tissue:
Expression of COUP-TFI or COUP-TFII was analyzed by immunohistochemistry in a human fetal heart (hFHs) at 12 weeks of gestation. Briefly, intact hearts were fixed with 4% PFA. Paraffin-embedded hearts were sectioned at 5 µm. Sections were mounted onto silane-coated slides, deparaffinized in xylene, rehydrated in graded ethanol series and washed in PBS. Serial sections were used for immunostaining of COUP-TFI (clone H8132, Perseus proteomics) or COUP-TFII (clone H7147, Perseus Proteomics) in combination with Troponin I (clone H-170, Santa Cruz). An additional hFH at 14 weeks of gestation was used to confirm findings observed in the 12 week-old hFH.

COUP-TFI and COUP-TFII were stained by indirect immunohistochemistry. After over night incubation with primary antibodies, slides were washed and incubated with biotin coupled Horse anti-mouse secondary antibody (Vector Labs) and 1.5% normal horse serum (Vector Labs) in PBS supplemented with 0.05% Tween-20. COUP-TF binding was visualized by using Streptavidin Alexa-488 (Molecular Probes, Life technologies) antibody.

ShRNA-mediated knockdown of COUP-TFs:
pLKO.1-lentiviral shRNA vectors targeting the human *COUP-TF I* and *II* genes were a kind gift from Dr A Beqqali, Academic Medical Center, Amsterdam. shRNA sequences are enclosed in supplemental table 7. A scrambled shRNA was used as control. Lentivirus was produced by co-transfection of the packaging vectors (pMD2.G, pRRE and pRSV/REV) with a shRNA target vector into HEK 293T cells (Thermo Scientific). For transfections, the construct DNA was mixed with Lipofectamine 2000 in Opti-MEM (Life Technologies) and was added to the media (DMEM containing 4% FCS). After 18 hours, cells were refreshed with DMEM-4% FCS. Lentiviruses were harvested 72 h after transfection and concentrated in a Beckman coulter ultracentrifuge at 20,000 rpm and at 4^0C for 2 hours. Lentiviruses were resuspended in 1% BSA-PBS and stored at -80°C. The titer was determined with a qPCR lentiviral titration kit (ABM Inc.).

EBs at day 17 were dissociated to single cells with 10x TryplE™ select (Life technologies). At day 20, cells were transduced with the lentiviral-shRNAs at an MOI of 50-100. For both *COUP-TF I* and *COUP-TF II*, five different target-specific shRNAs each, were tested. Two sequences that gave the most efficient knockdown were selected for further experiments. The expression of *COUP-TF I* and *II* following knockdown was evaluated by qPCR and Western blot analysis 7-10 days post transduction.

Transcription factor (TF) binding site analysis:
TF binding sites in the promoter regions of *KCNA5* and *KCNJ3* were analyzed using MatInspector (Genomatix, Munich, Germany) and weight matrices used for prediction

were described previously (1). MatInspector is accessible at http://www.genomatix.de/en/index.html and MatBase matrix library version 9.0 was used.

ChIP-qPCR assays:

ChIP assays were performed with Transcription ChIP kit (Diagenode) according to manufacturers instructions. $2*10^6$ cells from day 30 EBs derived from control or retinoic acid treated differentiations were used for each experiment. Intact EBs were washed twice with PBS and fixed for 10 min with 1% PFA followed by quenching with 0.125 M glycine. Samples were sonicated using Bioruptor (Diagenode) for 20 cycles of 30 sec ON/OFF each. Sheared chromatin was analysed on gel to ensure optimal sonication and a portion of total chromatin was set aside as input DNA. Protein A/G beads were used for immunoprecipitation (IP) carried out over night at 4°C. 10 µg of the following antibodies were used – COUP-TFI (Clone H8132, Perseus Proteomics); COUP-TFII (Clone H7147, Perseus Proteomics); Mouse IgG2a Isotype control (MAB003, R&D systems). After washing and elution of the beads, cross-links were reversed for 4 hours at 65°C. DNA was recovered by Phenol-Chloroform extraction.

For qPCR, samples were analyzed with promoter-specific primers and results are displayed as fold enrichment over mock. Results from three independent experiments were averaged and compared against IgG control. Primer sequences are enclosed (Table S11)

Western blot

Samples were separated on 8% SDS-polyacrylamide gels for 10 min at 90V and 110 min at 80V. Proteins were then transferred onto Immobilon-P PVDF membranes (Millipore, Bedford, MA) at 100V for 1.5 hours at 4°C. The membrane was blocked in 5% nonfat milk in TBS-Tween 20 for 1 hour at 4°C. Incubation was carried out overnight at 4°C in TBS-0.05% Tween 20 containing 2% nonfat milk with the following primary antibodies: COUP-TF I (1:500; Perseus proteomics); COUP-TF II (1:500; Perseus Proteomics); GAPDH (1:500; Millipore); Incubation with ACTN2 (1:2000, Sigma) was performed in TBS-0.05% Tween 20 containing 2% nonfat milk for 1 hour at room temperature. The corresponding secondary antibody conjugated to horseradish peroxidase (HRP) (1:2000; anti-mouse-HRP (#7076), Cell Signaling) was incubated for 1 h at room temperature in 2% nonfat milk. Blots were developed on X-ray films by using a chemiluminescent detection system (ECL; GE Life Science, Buckinghamshire, UK).

Drugs

The effects of drugs were tested in paired measurements 5 minutes after the onset of bath application. 4-AP was prepared as a 50 mmol/L stock solution in Tyrode's solution, and pH adjusted to 7.4, with the addition of HCl. CCh, a muscarinic receptor agonist, was prepared as a 10 mmol/L stock solution in Tyrode's solution. Nifedipine was prepared as a 10 mmol/L stock solution in DMSO and stored at 4 ºC. Stock solutions for the other compounds were prepared in DMSO: Vernakalant (30 mmol/L), XEN-D0101 (3 mmol/L), XEN-R0703 (2 mmol/L) and stored at-20^0C. All stock solutions were diluted appropriately before use.

Recombinant ion channel pharmacology

Studies were conducted at room temperature using the whole-cell patch-clamp technique. HEK293 cells expressing $K_{ir}3.1/3.4$, $K_{ir}2.1$, and CHO cells expressing $Na_v1.2$, $Ca_v1.2$ or hERG were used.

Maintenance of cell lines
HEK293 cells expressing Kir3.1/3.4 or Kir2.1, and CHO cell lines stably expressing hERG or Nav1.5 were maintained in media containing 10% FCS and appropriate selection antibiotic. Cells were grown either in suspension or in T-flasks and routinely passaged. Cells for patch clamping experiments were plated onto glass cover slips prior to use. Cells for automated patch clamping experiments were freshly prepared on each experimental day.

Cloned cardiac ion channel conventional electrophysiology
Standard gigaseal whole-cell patch-clamp techniques were performed at room temperature using glass pipettes (2-4 MΩ). HEKA EPC9/10 amplifiers and Pulse software were used for data acquisition. Series resistance was compensated by >70%. Voltage protocols were: Kir3.1/3.4, Kir2.1 (V_{Hold} -60mV, +60 mV/100 ms, ramp -140 mV/500 ms, -140 mV/100 ms, 0.1 Hz), hERG (V_{Hold} -80 mV, +20 mV/5s, -40 mV/5 s, 0.067 Hz).

Cloned cardiac ion channel automated electrophysiology
Experiments were performed using a QPatch48. For $Na_v1.5$ a train of 10 voltage clamp pulses (V_{Hold} -100mV, step -20mV / 20 ms, 1Hz) was appled to elicit inward Na^+ current. The train of 10 pulses was applied every minute for 4 minutes (4 x train) first in vehicle then again in presence of increasing concentrations of drug (1, 3 & 10 μM). Total duration of the cumulative concentration-response experiment was 16 minutes. Peak amplitude of the inward Nav1.5 current elicited by the tenth pulse of the fourth train in each condition was measured. Percentage inhibition of current by drug was calculated relative to current in vehicle.

Isolation of native human atrial myocytes
Studies reported here conform to the principles outlined in the Declaration of Helsinki, were reviewed and approved by the local research ethical approval committee (H03/035). Tissue was obtained from consenting patients (from Papworth Hospital NHS Trust, Cambridge, UK) and human atrial myocytes were mechano-enzymatically dissociated using a previously described protocol (2)

Dog rapid atrial pacing model of persistent AF
The *in vivo* anti-arrhythmic properties of XEN-R0703 were investigated in a canine RAP model of persistent AF. Animal experiments were carried out by CorDynamics, IL, USA, in compliance with the Guide for the Care and Use of Laboratory Animals (U.S.A.NIH publication No 85-23, revised 1985).

Briefly, male Beagles (10-15kg) were anaesthetized and ECG leads and bi-polar pacing leads were secured on the heart *via* a right thoracotomy under sterile conditions. Two bipolar pacing electrodes were attached to the right atrial appendage for pacing and recording of atrial effective refractory period (AERP). ECG electrodes were placed on the left ventricular epicardium near the ventricular apex (positive) and the musculature near the 9th-10th rib (negative). Wires were externalized in between the scapula. Animals recovered and 1-week post-surgery dogs were connected to an external pacemaker and the right atrium was paced at 400 bpm. After 2 weeks, conscious-dogs were placed in a sling and cardiac electrophysiology and AF inducibility were evaluated. ECGs were continuously recorded throughout the experiment via telemetry for all dogs during the dosing periods. For determination of atrial effective refractory period (AERP), hearts experienced cycles of 8 paced beats (3 Hz) of atrial origin (s1: at twice electrical diastolic threshold, using two discrete fixed cycle length) followed by an extra atrial stimulus (s2) delivered at varying coupling intervals from the eight beat train of s1. The coupling time that failed to elicit an s2 stimulus was noted as the AERP. AF inducibility was determined using three seconds of burst atrial pacing using 50 Hz pulses with a 2

ms duration at four times diastolic threshold current. The incidence of successful initiation over 25 attempts was recorded and percentage inducibility calculated as number of successful attempts divided by 25. AF inducibility greater than 25% was used as the arbiter to entry into the dosing protocol. Prior to electrophysiology experimentation, the conscious dog was placed in a sling restraint for the entire dosing period. Test article was administered in escalating doses every 60 minutes (15 minute loading period followed by a 45 minute maintenance dosing period). Atrial refractory period measurements were made at the end of loading-dose period. During the dose-maintenance period, AF inducibility testing was performed.

ECG Parameters Analysis
Measurements were assessed by two different investigators and compared to assure agreement. Van de Water's rate-corrected (QTc) interval of the ECG was taken as the mean value from 10-15 cardiac cycles at the end of the drug loading period.

References

1. Cartharius K, French K, Grote K, Klocke B, Haltmeier M, Klingenhoff A, Frisch M, Bayerlein M, Werner T. Bioinformatics. 2005; 21:2933-42.

2. Ford J, Milnes J, Wettwer E, Christ T. Human electrophysiological and pharmacological properties of XEN-D0101: A novel atrial selective Kv1. 5/I Kur inhibitor. J Cardiovasc Pharm 2013 61:408–415

Supplemental Tables:

Table S1: Gene lists represented in Venn diagrams of Fig. 2D-E. Please see enclosed excel file.

Table S2: Gene ontology terms and lists represented in Pie charts of Fig. 2F. Please see enclosed excel file.

Table S3: Detailed list of gene ontology terms and genes in hESC-VM and hESC-AM. Please see enclosed excel file.

Table S4: ShRNA sequences for COUP-TFI and COUP-TFII.

shRNA	Sequence	Match Region	NCBI Blast homology
Coup-TF I-sh#1	CGTCCGCAGGAACTTAACTTA	CDS	339-359 of CDS (1271 bp of NM_005654.4)
Coup-TF I-sh#2	CAGCTTCAACTGGCCTTACAT	CDS	1233-1253 of CDS (1271 bp of NM_005654.4)
Coup-TF I-sh#3	CCAGCCCAATCCAGGCCAGTA	CDS	492-512 of CDS (1271 bp of NM_005654.4)
Coup-TF I-sh#4	CTCTTCTTCGTCCGTTTGGTA	CDS	1162-1182 of CDS (1271 bp of NM_005654.4)
Coup-TF I-sh#5	GCCCAACAACATTATGGGCAT	CDS	627-647 of CDS (1271 bp of NM_005654.4)
Coup-TF II-sh#6	CGTGATTGATTCAGTATCTTA	3'UTR	1004-1024 of 3'UTR (2641 bp of NR2F2-001 ENST00000394166)
Coup-TF II-sh#7	GCCGTATATGGCAATTCAATA	CDS	1224-1244 of CDS (1244 bp of NM_021005.3)
Coup-TF II-sh#8	GTCGCCTTTATGGACCACATA	CDS	856-876 of CDS (1244 bp of NM_021005.3)
Coup-TF II-sh#9	CGGATATATTTCCCTGCTGTT	CDS	537-557 of CDS (1244 bp of NM_021005.3)
Coup-TF II-sh#10	CCTCCTCAGTCATAGAGCAAT	CDS	1121-1141 of CDS (1244 bp of NM_021005.3)

Table S5. Effect of I_{Kur} block (by 4-AP) and muscarinic receptor stimulation (by CCh) on hESC-atrial and hESC-ventricular CMs.

Group	Condition	RMP (mV)	APA_{max} (mV)	dV/dt_{max} (V/s)	APD_{20} (ms)	APD_{50} (ms)	APD_{90} (ms)	APA_{plat} (mV)
**hESC-AM (n=5)**	Basal	-71.7±1.9	79.7±3.7	26.3±2.7	20.8±3.7	44±10	145±21	62.2±9.0
	4-AP	-70.8±2.1	94.6±3.0*	33.4±3.5*	41.6±8.1*	72±13*	176±14*	86.4±6.5*
**hESC-VM (n=5)**	Basal	-69.2±2.5	98.7±3.8	50.4±10.8	82.0±15.9	132±21	181±30	96.8±6.0
	4-AP	-69.2±2.5	99.0±4.0	49.9±11.1	83.1±15.1	130±20	183±31	99.8±5.8
**hESC-AM (n=5)**	Basal	-71.6±2.2	85.8±2.2	31.9±2.3	30.2±4.8	75±6	160±16	68.7±2.1
	CCh	-76.4±2.1*	89.1±1.1	39.1±4.0*	25.4±3.8*	67±6*	156±19	72.6±2.3
**hESC-VM (n=5)**	Basal	-70.0±1.2	105.5±8.1	135.3±57	113.1±18.3	166±26	215±28	104.1±8.4
	CCh	-69.9±1.3	104.4±9.1	129.9±54	112.9±18.0	166±28	214±27	103.2±8.8

Mean±SEM. APs were measured at 1 Hz. *$P<0.05$ Paired Student's *t*-test.

AP = Action potential; 4-AP = 4-aminopyridine; APA_{max} = maximum AP amplitude; APA_{plat} = AP plateau amplitude; APD_{20}, APD_{50}, and APD_{90} = AP duration at 20, 50, and 90% repolarization, respectively; Basal = before addition of drug; CCh=Carbachol; dV/dt_{max} = maximum upstroke velocity; hESC-AM=hESC-atrial CMs; hESC-VM =hESC-ventricular CMs; RMP=resting membrane potential.

Table S6. Effect of Vernakalant on hESC-atrial and hESC-ventricular CMs.

Group	Condition	RMP (mV)	APA_{max} (mV)	dV/dt_{max} (V/s)	APD_{20} (ms)	APD_{50} (ms)	APD_{90} (ms)	APA_{plat} (mV)
hESC-AM (n=5)	Basal	-73.2±2.2	77.5±2.0	54.4±7.4	22.0±5.1	49.9±8.4	154.6±12.2	60.0±4.4
	Vernakalant	-74.2±2.5	80.3±2.2*	40.4±3.8*	29.7±4.0*	65.4±7.7*	177.2±08.2*	73.6±4.9*
hESC-VM (n=5)	Basal	-71.6±1.5	106±3.1	70.9±5.1	85.0±16.4	136.0±24.0	190.5±26.2	101.0±2.8
	Vernakalant	-71.2±1.6	104±2.2	58.3±6.1*	68.6±06.8	113.8±08.8	180.0±17.7	101.3±2.8

Mean±SEM. APs were measured at 1 Hz. *P<0.05 Paired Student's *t*-test.

AP = Action potential; APA_{max} = maximum AP amplitude; APA_{plat} = AP plateau amplitude; APD_{20}, APD_{50}, and APD_{90} = AP duration at 20, 50, and 90% repolarization, respectively; Basal = before addition of drug; dV/dt_{max} = maximum upstroke velocity; hESC-AM=hESC-atrial CMs; hESC-VM =hESC-ventricular CMs; RMP=resting membrane potential.

Table S7. Effect of XEN-D0101 on hESC-atrial and hESC-ventricular CMs.

Group	Condition	MDP (mV)	APA_{max} (mV)	dV/dt_{max} (V/s)	APD_{20} (ms)	APD_{50} (ms)	APD_{90} (ms)	APA_{plat} (mV)
**hESC-AM (n=5)**	Basal	-72.0±0.8	88.0±4.9	29.7±4.7	25.3±7.6	55.7±15.9	132.3±26.2	60.0±7.4
	XEN-D0101	-71.7±0.8	98.9±5.8*	37.9±6.1*	55.7±16.2*	91.3±22.6*	156.2±22.8*	86.8±7.4*
**hESC-VM (n=5)**	Basal	-72.9±1.0	104.3±4.6	40.7±11.0	93.0±15.0	153.3±15.3	233.6±20.4	101.5±5.5
	XEN-D0101	-73.0±1.0	105.3±5.8	39.4±10.7	101.3±14.0	158.4±18.0	237.4±21.9	103.7±6.5

Mean±SEM. APs were measured at 1 Hz. *P<0.05 Paired Student's *t*-test.

AP = Action potential; APA_{max} = maximum AP amplitude; APA_{plat} = AP plateau amplitude; APD_{20}, APD_{50}, and APD_{90} = AP duration at 20, 50, and 90% repolarization, respectively; Basal = before addition of drug; dV/dt_{max} = maximum upstroke velocity; hESC-AM=hESC-atrial CMs; hESC-VM =hESC-ventricular CMs; RMP=resting membrane potential.

Table S8. Summary of ion channel pharmacology of XEN-R0703

**Ion Channel**	**Platform**	**IC_{50}**	**K_{ir}3.1/3.4 Selectivity Ratio**
Kir3.1/3.4	CP	59 nM	-
hERG	CP	5.7µM	~100
Na_v1.5 (1Hz)	QPatch	19µM	>300
Nav1.5 (Tonic)	QPatch	22µM	~300
Ca_v1.2	Flex	30µM	~500
K_{ir}2.1	CP	>3µM	>>50
I_{KACh}	Myocyte /CP	81 % @ 300nM	

Recombinant ion channel pharmacology of XEN-R0703 was investigated using either conventional whole-cell patch-clamp (CP), automated electrophysiology using the Sophion QPatch platform (QP) or using a Ca^{2+} -sensitive dye, fluorescence-based assay (Flex). Native ion channel pharmacology was investigated using freshly dissociated human atrial myocytes and the conventional whole-cell patch-clamp technique.

Table S9. Effect of XEN-R0703 on hESC-atrial and hESC-ventricular CMs.

Group	Condition	RMP (mV)	APA_{max} (mV)	dV/dt_{max} (V/s)	APD_{20} (ms)	APD_{50} (ms)	APD_{90} (ms)	APA_{plat} (mV)
**hESC-AM (n=4)**	Basal	-72.6±2.6	84.9±2.6	33.5±5.2	27.9±5.3	75.6±7.8	148.5±13.3	68.8±2.8
	CCh	-77.1±2.5	88.9±1.4	40.0±5.2	23.9±4.6	68.1±7.7	141.2±12.4	72.4±3.1
	CCh+XEN-R0703	-71.3±3.0*	84.3±1.9*	35.2±4.8*	30.9±5.4*	77.4±9.4*	154.0±11.0*	72.9±3.0
**hESC-VM (n=5)**	Basal	-69.4±1.2	111.3±6.3	161.2±61.7	123.5±17.8	181.0±26.2	229.9±27.7	109.6±7.2
	CCh	-69.2±1.3	110.5±7.8	154.8±58.3	122.9±17.5	181.2±27.6	230.6±25.7	109.6±7.5
	CCh+XEN-R0703	-67.8±0.5	118.6±1.4	220.1±46.7	154.2±10.3	229.8±11.7	257.5±12.4	116.2±0.4

Mean±SEM. APs were measured at 1 Hz. *P<0.05 Paired Student's *t*-test.

AP = Action potential; APA_{max} = maximum AP amplitude; APA_{plat} = AP plateau amplitude; APD_{20}, APD_{50}, and APD_{90} = AP duration at 20, 50, and 90% repolarization, respectively; Basal = before addition of drug; dV/dt_{max} = maximum upstroke velocity; hESC-AM=hESC-atrial CMs; hESC-VM =hESC-ventricular CMs; RMP=resting membrane potential.

<u>Table S10:</u> Primer sequences used for qPCR assays.

TARGET	SEQUENCE
TNNT2 (TnTC)	F: TTCGACCTGCAGGAGAAGTT<br>R: GCGGGTCTTGGAGACTTTCT
NKX2.5	F: TTCCCGCCGCCCCCGCCTTCTAT<br>R: CGCTCCGCGTTGTCCGCCTCTGT
ACTN2	F: CTGCTGCTTTGGTGTCAGAG<br>R: TTCCTATGGGGTCATCCTTG
HAND2	F: ACATCGCCTACCTCATGGAC<br>R: TGGTTTTCTTGTCGTTGCTG
MYL7 (MLC2a)	F: CAGACCTGAAGGAGACCT<br>R: GTCAGCGTAAACAGTTGC
NPPA (ANF)	F: ACAGGATTGGAGCCCAGAG<br>R: GGAGCCTCTTGCAGTCTGTC
PITX2	F: AGCCATTCTTGCATAGCTCG<br>R: GTGTGGACCAACCTTACGGA
HAND1	F: AATCCTCTTCTCGACTGGGC<br>R: TGAACTCAAGAAGGCGGATG
HEY2	F: GATTCAGCCCTCCGAATG<br>R: TGGCAGAGAGGGACAAGAG
IRX4	F: TTCCGTTCTGAAGCGTGGTC<br>R:TGAAGCAGGCAATTATTGGTGT
MYL2 (MLC2v)	F: GATGTTCGCCGCCTTCCCCGC<br>R: GCAGCGAGCCCCCTCCTAGT
KCNA5 (K_V*1.5)*	F: CGAGGATGAGGGCTTCATTA<br>R: CTGAACTCAGGCAGGGTCTC
KCNJ3 (K_{ir}*3.1)*	F: AAAAACGATGACCCCAAAGA<br>R: TGTCGTCATCCTAGAAGGCA
KCNJ5 (Kir3.4)	F: GGACACCCCAGAAGTTAGCA<br>R: CCAATCTCCATGTCCTGGTT
COUP-TF I	F: AAGCCATCGTGCTGTTCAC<br>R: GCTCCTCAGGTACTCCTCCA
COUP-TF II	F: CCGAGTACAGCTGCCTCAA<br>R: TTTTCCTGCAAGCTTTCCAC

Table S11: Primer sequences used for ChIP-qPCR assay.

TARGET	SEQUENCE
KCNA5	F: CTGGTGTCTCCCGTCTCTGT<br>R: CAGGGCAACCAGATGAGAAT
KCNJ3	F:GCCTACTTATTTATTTGCTGTGTTGA<br>R: TCATCTGCAACAAAATCCAAA

Supplemental Figures:

Figure S1

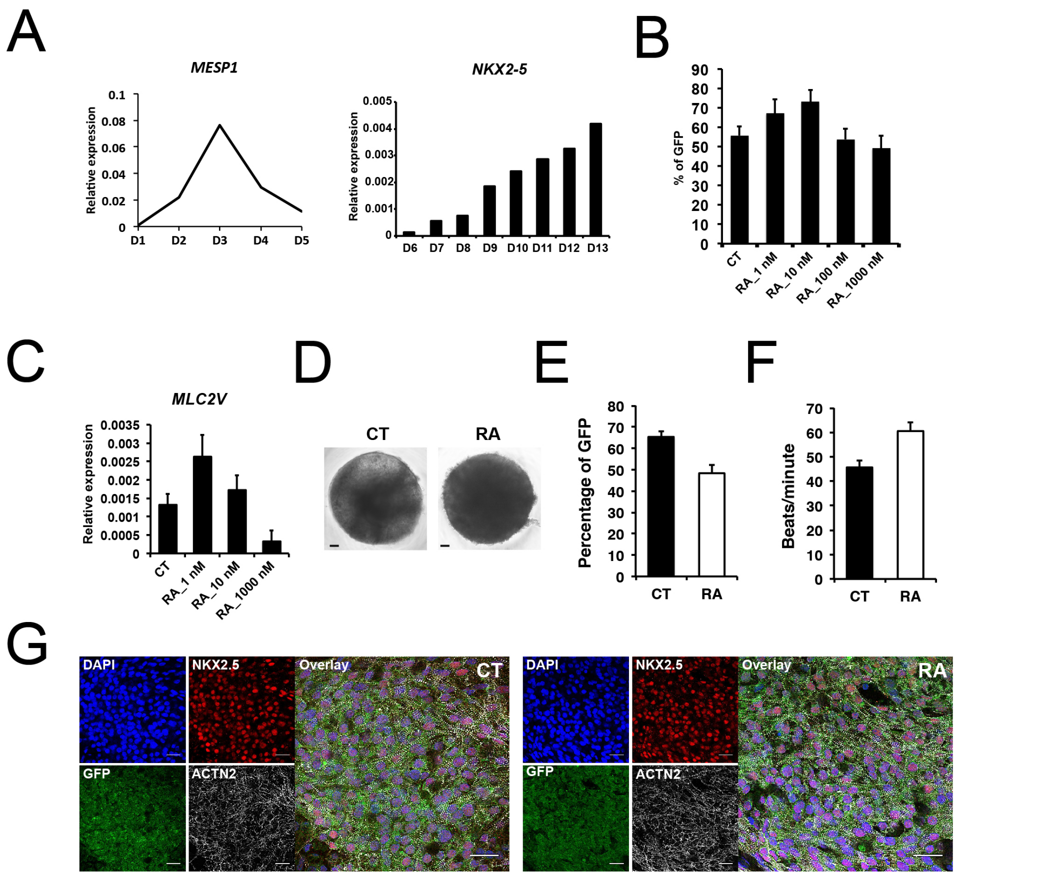

Figure S1: (A) mRNA expression of *MESP1* and *NKX2.5* during cardiac differentiation of hESCs. **(B)** Percentage of GFP expressing cells at day 15, resulting from differentiations treated with varying concentrations of RA from day 4-7. **(C)** mRNA expression of *MLC2V* in day 15 EBs, obtained from differentiations treated with varying concentrations of RA from day 4-7. (**D)** Morphology of embryoid bodies treated with DMSO (CT) or retinoic acid (RA) at day 6. Scale bar: 100 µm. **(E)** Bar graph demonstrating the percentage of GFP^{+} cells at day 15, obtained from three independent CT or RA differentiations. **(F)** Frequency of spontaneous contractions observed in CT or RA EBs at day 15. **(G)** Immunofluorescence for NKX2.5 and ACTN2 in GFP^{+} areas of CT (left) and RA (right) differentiations. Nuclei counterstained with DAPI, scale bars: 25 µm.

Figure S2:

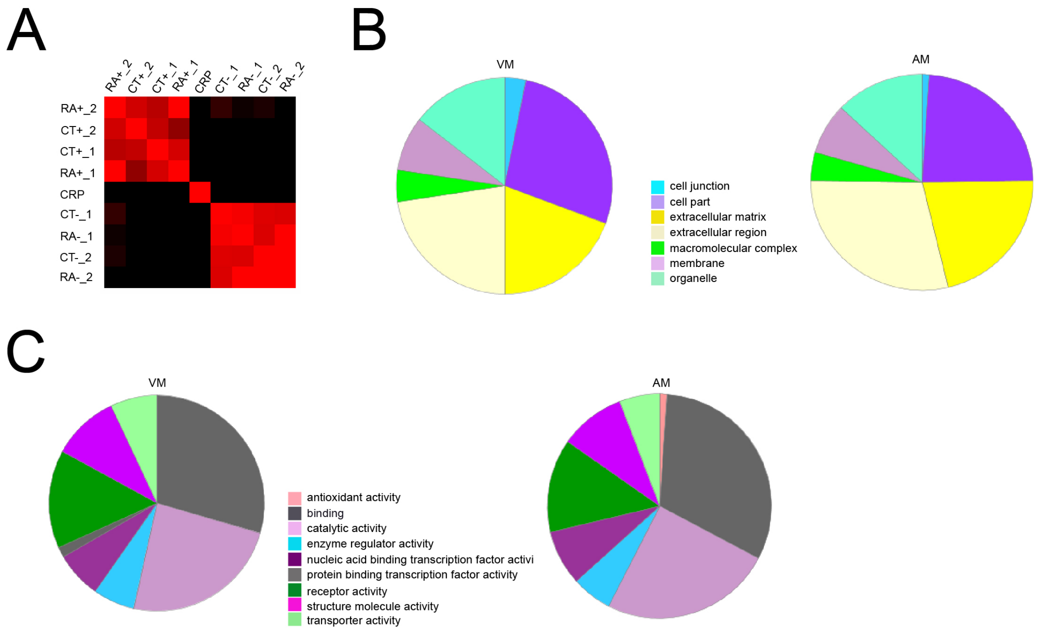

Figure S2: (A) Correlation heat map of GFP+ (CT+, RA+) and GFP- (CT-, RA) samples analyzed by microarray. **(B-C)** Pie chart illustrating the **(B)** cellular localization and **(C)** molecular functions of gene lists with increased expression in hESC-ventricular CMs (VM) and hESC-atrial CMs (AM).

Figure S3:

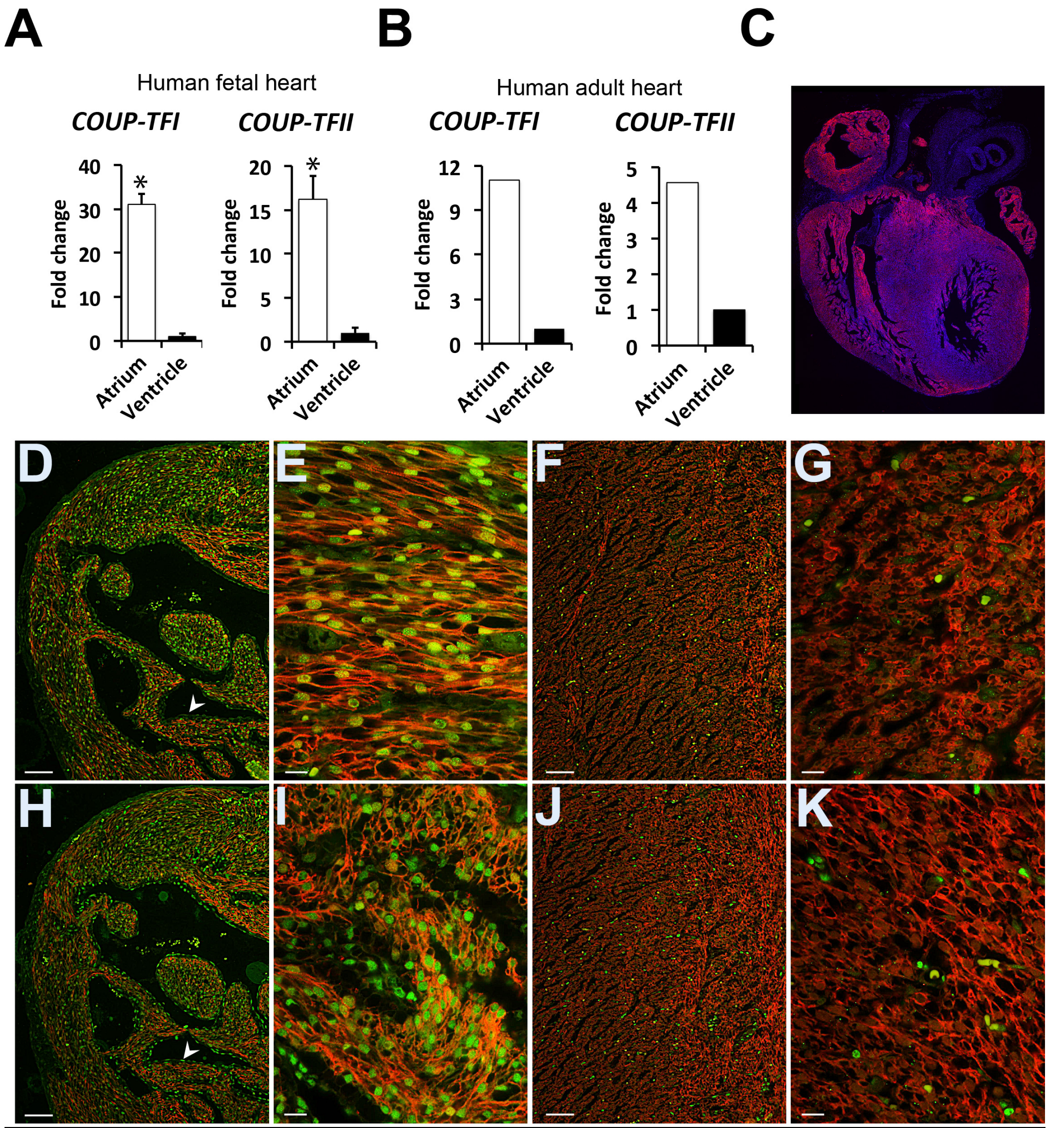

Figure S3. Expression of COUP-TFs is restricted to atrial chambers of the human heart. *COUP-TFI* and *COUP-TFII* mRNA expression in atria and ventricles of **(A)** human fetal hearts (n=3) and **(B)** human adult heart (n=1). **(C)** Overview of a 12 week-old fetal heart used for expression analysis, TNNI3 (Red) and DAPI (Blue). Immunofluorescence of COUP-TFI (Green) and TNNI3 (Red) in **(D-E)** atria and **(F-G)** ventricles of the fetal heart. COUP-TFII (Green) and TNNI3 (Red) immunofluorescence in **(H-I)** atria and **(J-K)** ventricles of the fetal heart. Arrows in D and H point to differences in endocardial expression of COUP-TFs. Scale bars:100 µm in panels D, F, H, J and 20 µm for E, G, I, K. *P<0.01 by Unpaired Student's t-test.

Figure S4:

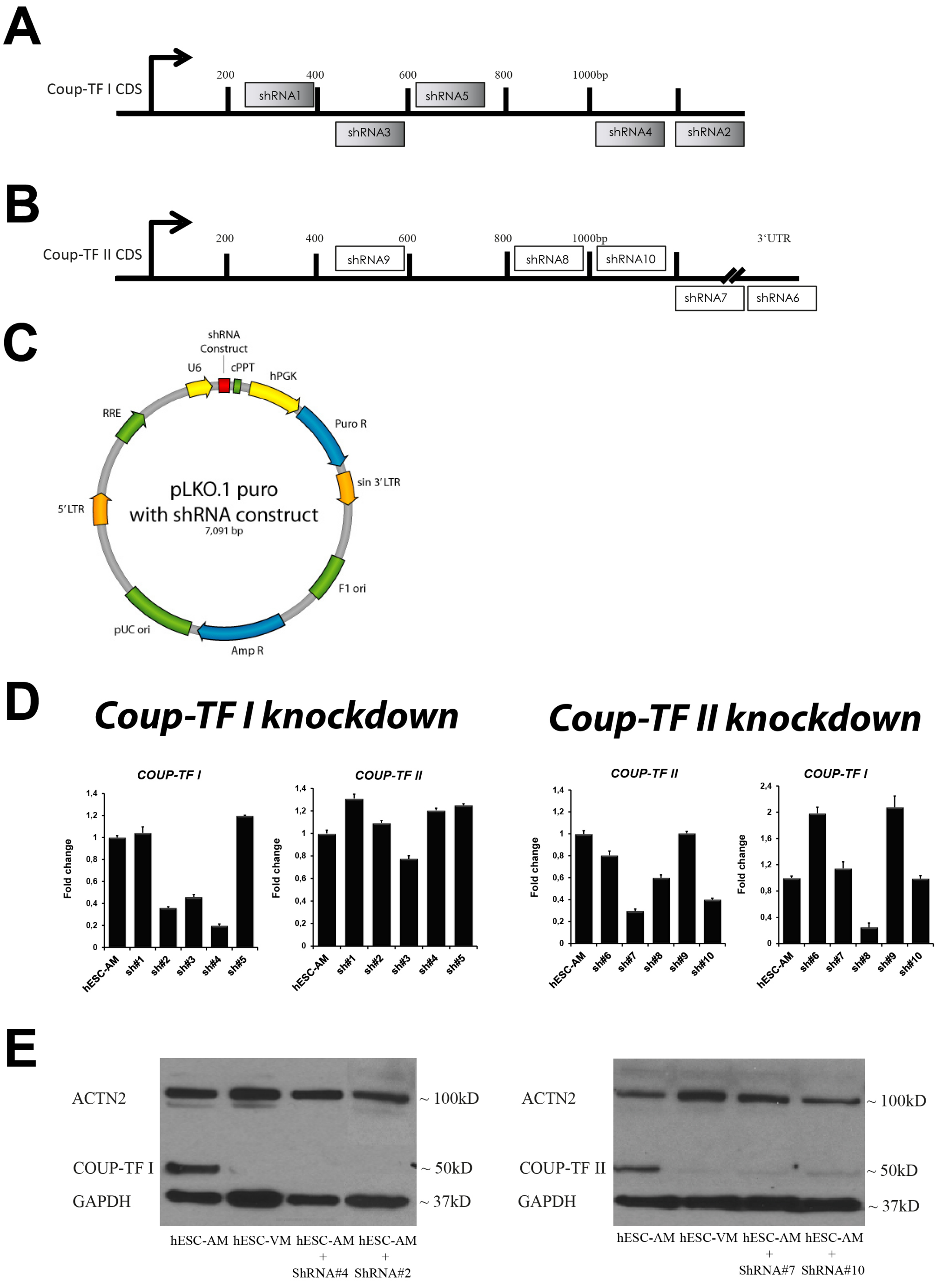

Figure S4: (A-B) Schematic illustrating **(A)** COUP-TFI-ShRNA or **(B)** COUP-TFII-ShRNA binding sites. **(C)** Schematic of the pLKO.1 vector. **(D)** mRNA expression of COUP-TFI and COUP-TFII following knockdown with five different shRNA constructs for COUP-TFI (left) and COUP-TFII (right). **(E)** Western blot analysis of COUP-TFI protein (left) and COUP-TFII protein (right) to confirm knockdown of COUP-TFI and COUP-TFII respectively.

Figure S5:

A

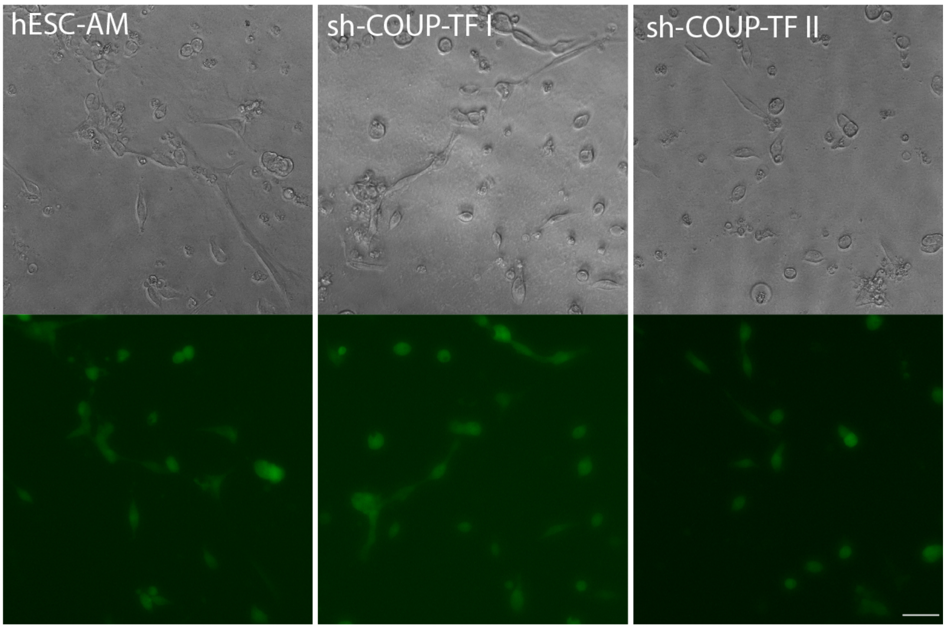

B

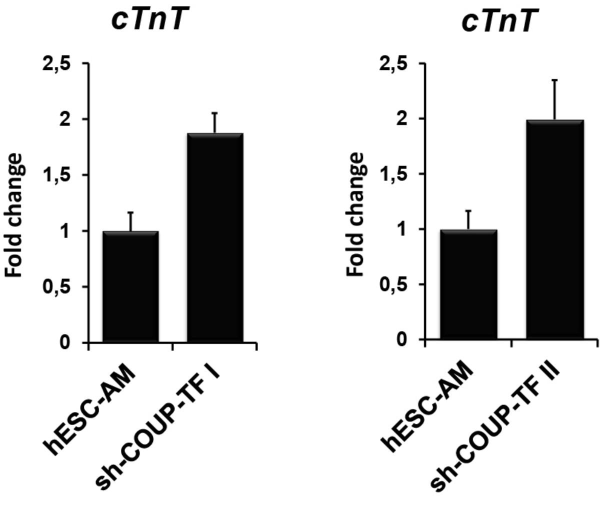

Figure S5: Expression of **(A)** GFP or **(B)** cTnT in hESC-atrial CMs (hESC-AM) compared with COUP-TFI (left) or COUP-TFII (right) knockdown. Scalebar: 100 uM.

Figure S6: Genomatix-defined NR2F matrix family

**V$NR2F**	Nuclear receptor subfamily 2 factors	V$ARP1.01	Apolipoprotein AI regulatory protein 1, NR2F2, DR1 sites
		V$COUP.01	Chicken ovalbumin upstream promoter 1 (COUP-TFI) and chicken ovalbumin upstream promoter 2 (COUP-TFII), DR1 sites
		V$COUP.02	Chicken ovalbumin upstream promoter (COUP-TF), DR0 sites
		V$HNF4.01	Hepatic nuclear factor 4, DR1 sites
		V$HNF4.02	Hepatic nuclear factor 4, DR2 sites
		V$HNF4.03	Hepatic nuclear factor 4, DR1 sites
		V$HNF4A.01	Hepatic nuclear factor 4alpha, DR1 sites
		V$HPF1.01	HepG2-specific P450 2C factor-1, DR1 sites
		V$PNR.01	Photoreceptor-specific nuclear receptor subfamily 2, group E, member 3 (Nr2e3), DR1 sites
		V$TR2.01	Nuclear hormone receptor TR2, DR5 binding sites
		V$TR2_TR4.01	DR1 binding sites for TR2 homodimers or TR2/TR4 heterodimers
		V$TR4.01	TR4 homodimer, DR1 site
		V$TR4.02	TR4 homodimer, DR1 site

<u>Figure S7:</u>

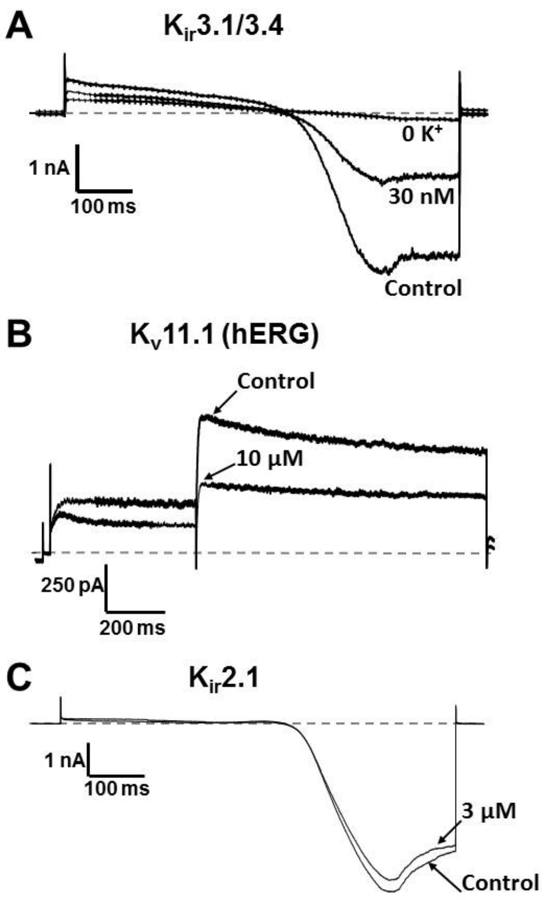

<u>**Figure S7:**</u> **(A)** Effect of XEN-R0703 on recombinant Kir3.1/3.4 channel expressed in HEK293 cells. Original steady-state current trace are shown in the absence (Control) and presence of 30 nM XEN-R0703 and then following removal of extracellular K^+ (replaced by equimolar Na^+, '0K') to assess passive leak. **(B)** Effect of 10 μM XEN-R0703 on recombinant Kv11.1 (hERG) channel expressed in HEK293 cells. Original steady-state current trace are shown the in absence and presence of XEN-R0703. **(C)** Effect of XEN-R0703 on recombinant Kir2.1 channel expressed in HEK293 cells. Original steady-state current trace are shown the in absence and presence of 3 μM XEN-R0703 and then following removal of extracellular K^+ (replaced by equimolar Na^+) to assess passive leak. The voltage protocol and conditions for each assay are given in the methods

Figure S8:

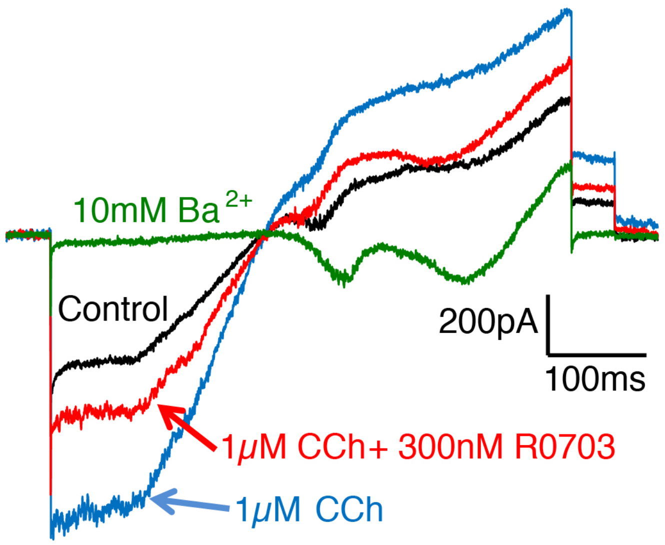

Figure S8: Effect of XEN-R0703 on native human cardiac I_{KACh}. Whole-cell patch-clamp recordings were made from freshly dissociated human atrial cardiomyocytes (*N*=3). Original control current traces are shown before and after the application of 1 μM CCh to activate cardiac I_{KACh}. 300 nM XEN-R0703 was then applied in the presence of 1 μM CCh. At the end of the experiment 10 mM $BaCl_2$ was applied to fully inhibit both inward rectifier potassium currents, I_{K1} and I_{KACh}.
